# Nucleolar activity and CENP-C regulate CENP-A and CAL1 availability for centromere assembly in meiosis

**DOI:** 10.1242/dev.130625

**Published:** 2016-04-15

**Authors:** Lucretia Kwenda, Caitriona M. Collins, Anna A. Dattoli, Elaine M. Dunleavy

**Affiliations:** Centre for Chromosome Biology, Biomedical Sciences, National University of Ireland Galway, Galway, Ireland

**Keywords:** Centromere, Meiosis, Spermatogenesis, CENP-A, CID, Nucleolus

## Abstract

The centromere-specific histone CENP-A is the key epigenetic determinant of centromere identity. Whereas most histones are removed from mature sperm, CENP-A is retained to mark paternal centromeres. In *Drosophila* males we show that the centromere assembly factors CAL1 and CENP-C are required for meiotic chromosome segregation, CENP-A assembly and maintenance on sperm, as well as fertility. In meiosis, CENP-A accumulates with CAL1 in nucleoli. Furthermore, we show that CENP-C normally limits the release of CAL1 and CENP-A from nucleoli for proper centromere assembly in meiotic prophase I. Finally, we show that RNA polymerase I transcription is required for efficient CENP-A assembly in meiosis, as well as centromere tethering to nucleoli.

## Abstract (precis)

**Summary:** Novel roles are uncovered for centromere assembly factors CENP-C and CAL1 in meiotic chromosome segregation, CENP-A assembly and maintenance of sperm, as well as fertility in *Drosophila* males.

## INTRODUCTION

Centromeres are chromosomal loci where microtubules attach to ensure proper chromosome segregation at cell division. Centromeres are not determined by DNA sequence but instead are specified epigenetically by the centromere-specific histone CENP-A (Allshire and Karpen, 2008; McKinley and Cheeseman, 2015; Mendiburo et al., 2011). Each cell cycle, CENP-A is assembled and maintained to ensure centromere propagation (Valente et al., 2012). Moreover, centromere identity must be marked on gametes; whereas the majority of histones are removed from mature sperm, CENP-A is among the few histones retained, marking centromere identity on paternal chromosomes (Palmer et al., 1990; Raychaudhuri et al., 2012).

In the last decade, studies of the cell cycle timing of CENP-A assembly and the discovery of CENP-A-specific assembly factors have provided great insights into the mechanisms of CENP-A assembly in mitosis (Dunleavy et al., 2009; Foltz et al., 2009; Müller and Almouzni, 2014). In most organisms, mitotic CENP-A assembly occurs at late M and early G1 phase of the cell cycle (Chen et al., 2014; Jansen et al., 2007; Nechemia-Arbely et al., 2012; Schuh et al., 2007). Mechanisms of meiotic CENP-A assembly are now emerging and differ from those of mitosis. In *Drosophila* males, CENP-A [also known as Centromere identifier (CID) – FlyBase] is assembled at meiotic prophase I and again during spermatid differentiation (Dunleavy et al., 2012; Raychaudhuri et al., 2012). In plants, CENP-A is assembled in prophase I (Schubert et al., 2014). Worms show unusual meiotic CENP-A dynamics; CENP-A is removed and re-assembled in prophase I (Monen et al., 2005). Investigations into requirements for meiotic CENP-A assembly using RNAi approaches in fly testes implicate the mitotic CENP-A assembly factors Centromeric protein-C (CENP-C) and Chromosome alignment defect 1 (CAL1) (Dunleavy et al., 2012; Raychaudhuri et al., 2012). Yet, given differences in the assembly timing between meiosis and mitosis, the mechanisms by which CENP-C and CAL1 assemble meiotic CENP-A might be novel. Furthermore, CAL1 and CENP-C show unexpected localisation dynamics in meiosis; in fly spermatocytes centromeric CAL1 is not detectable past the first phase of CENP-A assembly (prophase I), while centromeric CENP-C is reduced prior to the second phase of CENP-A assembly (Dunleavy et al., 2012; Raychaudhuri et al., 2012). More recently, *Drosophila* mutants for *Cenp-C* and *cal1* have uncovered roles for CENP-C and CAL1 in centromere clustering and pairing in female meiosis (Unhavaithaya and Orr-Weaver, 2013), highlighting potential specific roles in meiosis.

Accumulating evidence suggests functional interplay between centromeres and nucleoli, the nuclear sites of rDNA transcription. First, centromeres are often positioned at the periphery of nucleoli in cultured cells (Guttenbach et al., 1996; Padeken et al., 2013) and the association has been functionally linked to chromatin silencing and genome stability (Padeken et al., 2013). Second, the key centromere assembly factor CAL1 and its functional human homologue Holliday junction recognition protein (HJURP), as well as human CENP-C (CENPC), localise to both centromeres and nucleoli (Dunleavy et al., 2009; Erhardt et al., 2008; Foltz et al., 2009; Pluta and Earnshaw, 1996; Wong et al., 2007). Yet the function of nucleolar CAL1/HJURP or CENP-C is not known. Centromere positioning at nucleoli has also been linked to meiotic chromosome segregation (Unhavaithaya and Orr-Weaver, 2013). However, whether centromere positioning is connected to CENP-A assembly in mitosis or meiosis has not been explored. Third, nucleolar proteins associate with CENP-A in mitotic cells (Dunleavy et al., 2009; Foltz et al., 2009, 2006). In flies, Nucleoplasmin (NLP) localises to centromeres and is required for centromere clustering at nucleoli (Padeken et al., 2013), while Modulo (nucleolin in mammals) interacts with CAL1 and is required for newly synthesized CAL1 and CENP-A localisation to centromeres (Chen et al., 2012). However, knowledge of nucleolar proteins involved in meiotic CENP-A assembly is currently lacking. Last, nucleolar transcription has also been implicated in CENP-A assembly in mitosis (Chan and Wong, 2012; Wong et al., 2007), but requirements in meiotic CENP-A assembly have not been investigated.

Using *Cenp-C* and *cal1* mutants, we uncover specific roles for CENP-C and CAL1 in centromere assembly, maintenance and function in male meiosis and spermatogenesis in *Drosophila*. We reveal a novel role for nucleoli as storage hubs for CENP-A and CAL1 prior to centromere assembly in meiotic prophase I.

## RESULTS

### CENP-C and CAL1 are required for chromosome segregation in male meiosis and in fertility

**Fig. 1.**
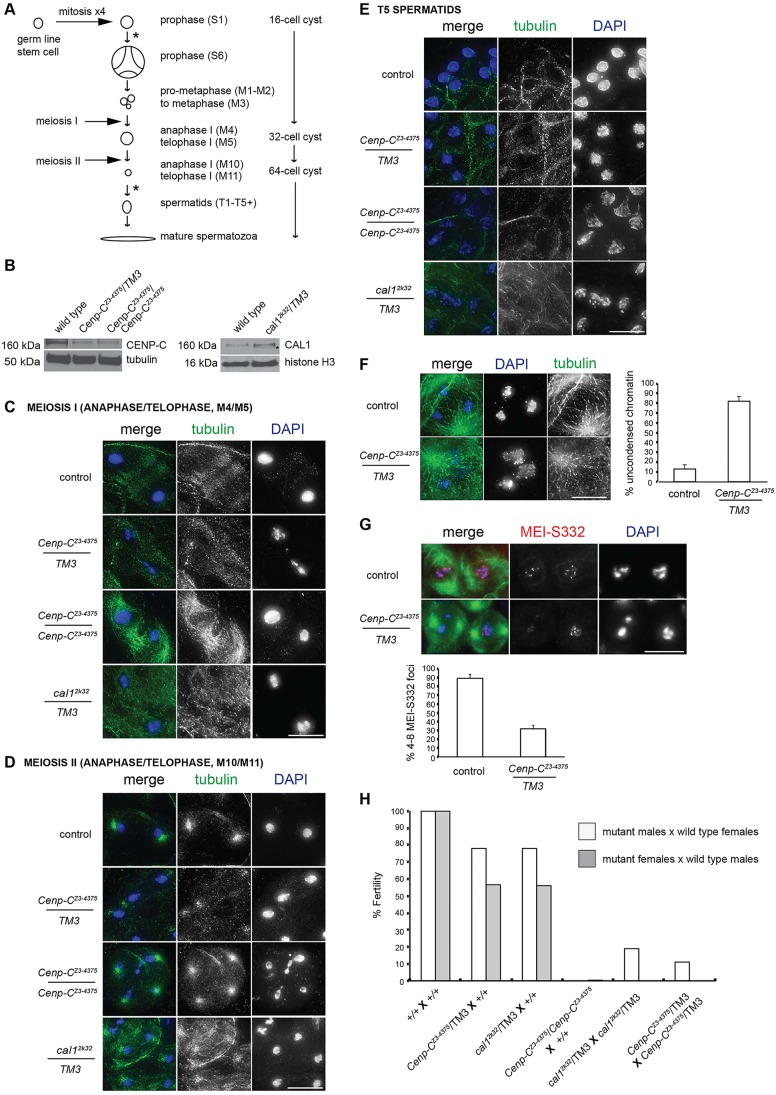
***Cenp-C^Z3-4375^* and *cal1^2k32^* mutants are defective in male meiosis and fertility.** (A) Schematic of *Drosophila* male meiosis and spermatogenesis (see main text). Asterisks mark the two phases of meiotic CENP-A assembly. (B) Western analysis of fractionated larval testes extracts. (Left) Wild type and *Cenp-C^Z3-4375^* heterozygotes and homozygotes probed with anti-CENP-C antibody. (Right) Wild type and *cal1^2k32^* heterozygotes probed with anti-CAL1 antibody. Loading controls are tubulin and histone H3. (C,D) Meiosis I (M4/M5) cells (C) and meiosis II (M10/M11) cells (D) from control, *Cenp-C^Z3-4375^* and *cal1^2k32^* testes fixed and stained with antibodies against tubulin (green). DNA is stained with DAPI (blue). (E) T5 spermatids from control, *Cenp-C^Z3-4375^* and *cal1^2k32^* testes fixed and stained for tubulin and DNA. (F) (Left) Control and *Cenp-C^Z3-4375^/TM3* prometaphase I (M1a) spermatocytes fixed and stained for tubulin and DNA. (Right) Quantitation of control and *Cenp-C^Z3-4375^/TM3* M1a spermatocytes with uncondensed chromatin (*n*=100, three replicates). Error bars indicate s.d. (G) (Top) Control and *Cenp-C^Z3-4375^/TM3* prometaphase/metaphase I (M2/M3) spermatocytes fixed and stained with antibodies against MEI-S332 (red), tubulin (green) and for DNA (blue). (Bottom) Quantitation of control and *Cenp-C^Z3-4375^/TM3* M2/M3 spermatocytes with four to eight MEI-S332 foci (*n*=100, three replicates). Error bars indicate s.d. (H) Fertility test for *Cenp-C^Z3-4375^* and *cal1^2k32^* males and females of the indicated genotypes; 100% fertility equates to the total number of eclosed adults after 10 days when wild-type (*+/+*) males are crossed to wild-type females (*n*=500). Scale bars: 10 µm.

In *Drosophila* female meiosis, *Cenp-C^Z3-4375^* and *cal1^2k32^* alleles are defective in centromere clustering and pairing, as well as chromosome segregation (Unhavaithaya and Orr-Weaver, 2013). *Cenp-C^Z3-4375^* is a homozygous viable, C-terminal missense mutation, whereas *cal1^2k32^* truncates CAL1 and is homozygous lethal (Unhavaithaya and Orr-Weaver, 2013). We tested if *Cenp-C^Z3-4375^* and *cal1^2k32^* mutants are defective in male meiosis. Meiotic stages are easily distinguished in *Drosophila* as spermatocytes develop sequentially in cysts and have been precisely staged (Cenci et al., 1994; Fuller, 1993). In brief, one germ line stem cell undergoes four mitoses to generate a cyst of 16 primary spermatocytes, which enter meiosis I (S1-S6, M1-M3) and divide to generate a 32-cell cyst of secondary spermatocytes (M4-M9), which undergo meiosis II to generate a 64-cell cyst (M10) that differentiates as spermatids (T1-T5+) into 64 mature spermatozoa (Cenci et al., 1994) (Fig. 1A).

To determine CENP-C or CAL1 protein levels in *Cenp-C^Z3-4375^* or *cal1^2k32^* male germ cells, we prepared nuclear soluble and chromatin-bound extracts from larval testes and performed western blotting for CENP-C or CAL1 (Fig. 1B). In *Cenp-C^Z3-4375^* heterozygotes and homozygotes, we detected full-length CENP-C in the nuclear soluble fraction, but levels were at least one-third reduced compared with the wild-type control, indicating a partial destabilisation of CENP-C, as reported for *Cenp-C^Z3-4375^* oocytes (Unhavaithaya and Orr-Weaver, 2013). In *cal1^2k32^* heterozygotes, we detected full-length CAL1 in the chromatin fraction at a level comparable to wild type, as well as truncated CAL1 (Fig. 1B). Staining of testes from *Cenp-C^Z3-4375^* or *cal1^2k32^* mutants with antibodies against tubulin to mark spindles and with DAPI to mark DNA revealed meiotic chromosome segregation defects, as well as abnormal spermatids (Fig. 1C-E, Table 1). Segregation defects were rarely observed in control (wild-type or *+/TM3*) meiosis I or II cysts, and T5 spermatids were mostly normal in morphology (Table 1). *Cenp-C^Z3-4375^* mutants showed segregation defects in meiosis I (36-38%) and II (12-16%), and T5 spermatid nuclei were abnormally decondensed (30-50%). Notably, *Cenp-C^Z3-4375^* homozygotes displayed a high frequency of anaphase bridges in meiosis I, and meiosis II was less perturbed compared with heterozygotes, suggesting that CENP-C is particularly important for meiosis I. Expression of an extra copy of YFP-tagged CENP-C in *Cenp-C^Z3-4375^* mutants reduced the frequency of defects and anaphase bridges were not observed (Table 1). In *cal1^2k32^* testes, meiosis I spermatocytes were rare, perhaps due to a developmental arrest or delay, and perturbed (57.1%), while 77.3% of meiosis II divisions were perturbed, mostly in nuclear shape. In 80% of *cal1^2k32^* spermatids nuclei were highly decondensed, stretched or fragmented (Fig. 1E) and the expression of an extra copy of GFP-tagged CAL1 restored normal spermatid morphology in adults (Fig. S1A). Quantitation of tubulin staining in *Cenp-C^Z3-4375^/TM3* and *cal1^2k32^*/*TM3* spermatocytes revealed that both mutants had defective spindle morphologies in meiosis I and II compared with wild type (Fig. S1B), which was likely to be due to roles of CENP-C and CAL1 in centromere/kinetochore assembly.

**Table 1.**
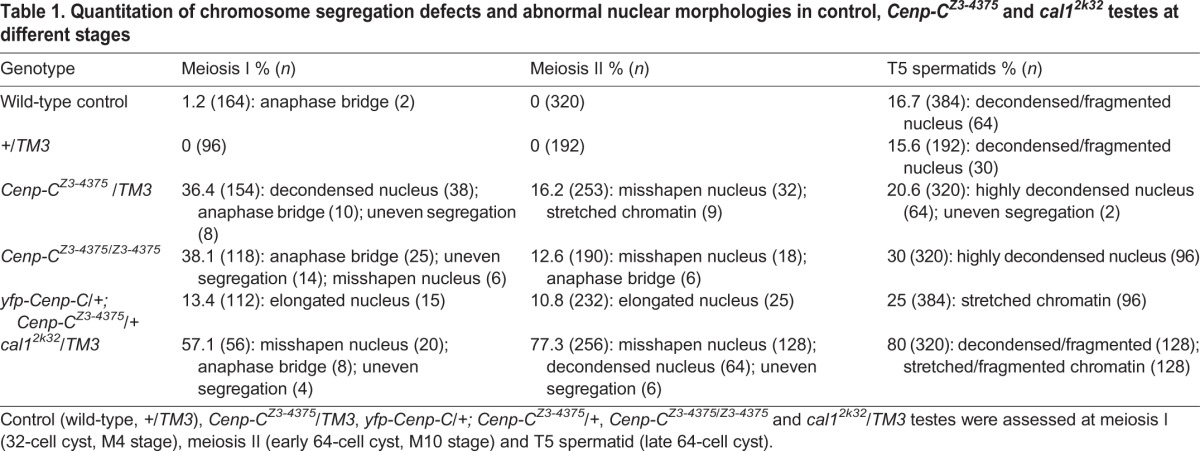
**Quantitation of chromosome segregation defects and abnormal nuclear morphologies in control, *Cenp-C^Z3-4375^* and *cal1^2k32^* testes at different stages**

Unexpectedly, *Cenp-C^Z3-4375^/TM3* spermatocytes also displayed uncondensed chromatin at prometaphase I (M1a/b) (Fig. 1F). At this stage, three chromosome territories are normally visible, corresponding to the three large chromosome pairs in *Drosophila* (Cenci et al., 1994). Yet in 80% of *Cenp-C^Z3-4375^/TM3* spermatocytes the chromosomes were not condensed at this stage compared with 10% of control cells (Fig. 1F). Next, we stained *Cenp-C^Z3-4375^/TM3* testes with an antibody against MEI-S332, a marker of sister chromatid cohesion (Tang et al., 1998) that is enriched at centromeres at prometaphase to anaphase I to prevent the segregation of sister chromatids until meiosis II (Nogueira et al., 2014). Staining of control late prometaphase/metaphase I (M2/M3) spermatocytes typically showed four pairs of spots in ∼90% of cells (Fig. 1G). By contrast, the number of *Cenp-C^Z3-4375^/TM3* spermatocytes that progressed to M2/M3 with detectable MEI-S332 foci was ∼30% (Fig. 1G), indicating a reduction or premature loss of sister chromatid cohesion.

Finally, we tested if meiotic chromosomal defects observed in *Cenp-C^Z3-4375^* or *cal1^2k32^* mutants might correlate with reduced fertility in males (Fig. 1H), in line with a report in humans (Dong et al., 2012). As reported for *Cenp-C^Z3-4375^* homozygous females (Unhavaithaya and Orr-Weaver, 2013), we found that *Cenp-C^Z3-4375^* homozygous males are also sterile, and fertility tests revealed that *Cenp-C^Z3-4375^/TM3* males mated with wild-type or *Cenp-C^Z3-4375^/TM3* females gave rise to reduced numbers of adults compared with control crosses. Similarly, *cal1^2k32^/TM3* males mated with wild-type or *cal1^2k32^/TM3* females gave rise to fewer adults than controls. Reciprocal tests with *Cenp-C^Z3-4375^* or *cal1^2k32^* females crossed to wild-type males revealed that fertility was even further reduced in females. These results indicate that CAL1 and CENP-C impact both male and female fertility. We conclude that *Cenp-C^Z3-4375^* and *cal1^2k32^* mutations negatively affect chromosome segregation in male meiosis I and II, as well as fertility in both sexes.

### CENP-C and CAL1 are required for CENP-A assembly/maintenance in male meiosis and spermatogenesis

**Fig. 2.**
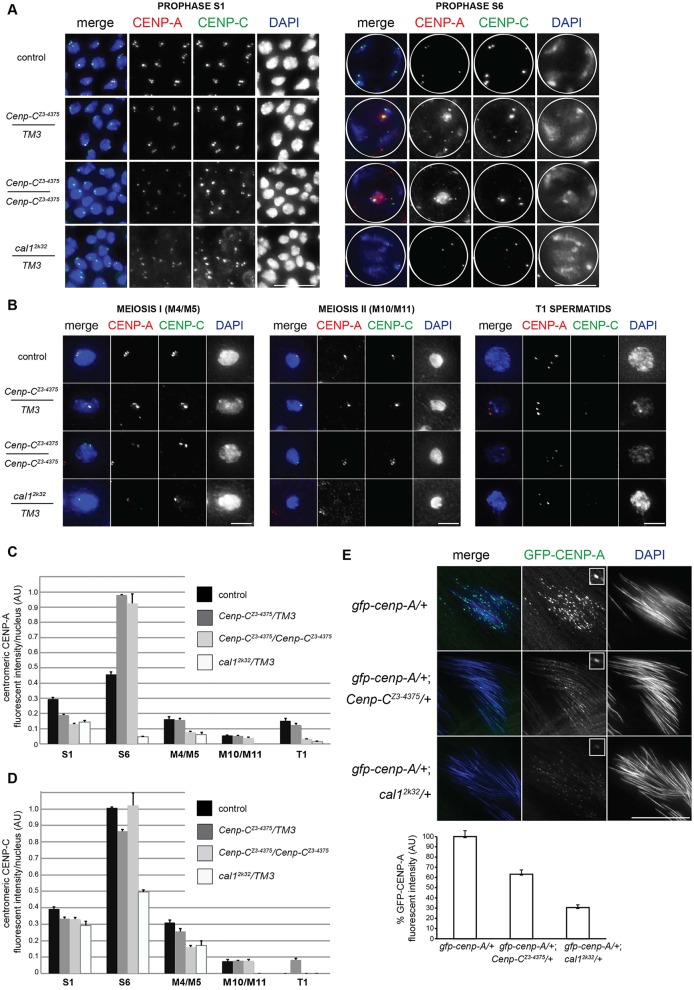
***Cenp-C^Z3-4375^* and *cal1^2K32^* mutants are defective in meiotic CENP-A assembly and maintenance.** (A) Prophase I nuclei at S1 (left) and S6 (right) from control, *Cenp-C^Z3-4375^* and *cal1^2k32^* testes were fixed and immunostained for CENP-A (red) and CENP-C (green); DNA is stained with DAPI (blue). White circle outlines S6 nuclei. (B) Meiosis I (M4/M5,) meiosis II (M10/M11) and T1 nuclei from control, *Cenp-C^Z3-4375^* and *cal1^2k32^* testes fixed and stained for CENP-A (red), CENP-C (green) and DNA. (C,D) Quantitation of total centromeric CENP-A (C) or CENP-C (D) (fluorescence intensity/nucleus) at S1 (*n*=30 each), S6 (*n*=30 each), M4/M5 (*n*=50 each), M10/M11 (*n*=50 each) and T1 (*n*=50 each) in control, *Cenp-C^Z3-4375^* and *cal1^2k32^* testes. Error bars indicate s.e.m. (E) (Top) Mature sperm bundles from *gfp-cenp-A/+, gfp-cenp-A/+; Cenp-C^Z3-4375^* or *gfp-cenp-A/+; cal1^2K32^* flies (green) fixed and stained with DAPI (grey). Insets show a single enlarged centromere. (Bottom) Quantitation of centromeric GFP-CENP-A fluorescence intensity in control, *Cenp-C^Z3-4375^* and *cal1^2k32^* lines expressing GFP-tagged CENP-A (*n*=300 centromeres). Error bars indicate s.e.m. Scale bars: 15 µm in A,E; 5 µm in B.

CENP-C and CAL1 play major roles in mitotic centromere assembly (Erhardt et al., 2008). Previous RNAi experiments showed that depletion of CENP-C or CAL1 in testes reduces meiotic CENP-A assembly (Dunleavy et al., 2012; Raychaudhuri et al., 2012). We next investigated whether *Cenp-C* or *cal1* mutations impact CENP-A assembly in male meiosis. We focused our analysis on the two phases of meiotic CENP-A assembly (Dunleavy et al., 2012; Raychaudhuri et al., 2012): (1) prophase I (between S1 and S6); and (2) on spermatids (between M10 and T1). We fixed and stained *Cenp-C* or *cal1* mutant testes with antibodies against CENP-A and CENP-C and quantified total centromeric CENP-A or CENP-C intensity per nucleus at S1, S6, M4/M5, M10/M11 and T1 stages (Fig. 2).

As expected for wild-type control spermatocytes, CENP-A intensity increased between S1 and S6, indicating CENP-A assembly in prophase I (Fig. 2A,C). CENP-A intensity dropped by approximately half between S6 and M4/M5, and again by half between M4/M5 and M10/M11, reflecting two rounds of chromosome segregation without CENP-A assembly (Fig. 2B,C). Finally, CENP-A intensity increased again between M10/M11 and T1, indicating the second phase of CENP-A assembly (Fig. 2B,C). CENP-C followed similar assembly dynamics to CENP-A in wild-type spermatocytes, except that CENP-C is not detected on T1 spermatids (Fig. 2D).

For *Cenp-C^Z3-4375^* heterozygotes, although starting levels of CENP-A and CENP-C at S1 were reduced compared with wild type, their intensity increased dramatically between S1 and S6, indicating prophase I CENP-A and CENP-C assembly (Fig. 2A,D). Between S6 and M4/M5, the intensity of both CENP-A and CENP-C dropped to as little as one-fifth, and halved between M4/M5 and M10/M11 (Fig. 2B,D). CENP-A intensity increased significantly (*P*<0.01, *t*-test) between M10/M11 and T1, indicating some CENP-A assembly, while CENP-C, which is usually removed at the T1 stage, was detected (Fig. 2B,D). In *Cenp-C^Z3-4375^* homozygotes, CENP-A and CENP-C assembly occurred in prophase I; however, CENP-A and CENP-C intensity was reduced at M4/M5 and M10/M11 compared with wild type and *Cenp-C^Z3-4375^* heterozygotes, and no significant CENP-A assembly was detected on T1 spermatids (*P*=0.78, *t*-test). For *cal1^2k32^* mutants, no CENP-A assembly was observed in prophase I and CENP-A intensity remained low throughout meiosis I and II, with low CENP-A assembly (10% of control level) at T1 (Fig. 2C). Despite some CENP-C assembly in prophase I, CENP-C levels remained low for the duration of meiosis in *cal1^2k32^* mutants (Fig. 2A,B,D), as confirmed by western analysis of testes extracts (Fig. S2A). Introduction of YFP-tagged CENP-C into *Cenp-C^Z3-4375^* mutants or GFP-tagged CAL1 into *cal1^2k32^* mutants restored CENP-A levels in prophase I (Fig. S2B), indicating that the centromere assembly defects are due to the lack of CAL1 or CENP-C.

Next we tested whether CENP-C and CAL1 are required for CENP-A maintenance on mature sperm, after most other histones are replaced by protamines (Dunleavy et al., 2012; Palmer et al., 1990; Raychaudhuri et al., 2012). As CENP-A antibody penetration is poor on mature sperm, to visualise its localisation we expressed a GFP-tagged CENP-A in *Cenp-C^Z3-4375^* and *cal1^2k32^* heterozygotes, then fixed and stained adult testes for DNA to identify mature ‘needle-like’ sperm bundles. Strikingly, we observed reduced centromeric GFP-CENP-A on mature sperm in *Cenp-C^Z3-4375^* and *cal1^2k32^* heterozygotes (to 63.3% and 30.4%, respectively) compared with controls (Fig. 2E). Taken together, these results demonstrate a role for CENP-C and CAL1 in CENP-A and/or CENP-C assembly and maintenance in meiosis, as well as in CENP-A maintenance on mature sperm.

### Meiotic CENP-A accumulates in nucleoli in prophase I

Observations of enhanced CENP-A assembly in prophase I in *Cenp-C^Z3-4375^* mutants (Fig. 2C) and increased levels of chromatin-bound CENP-A (Fig. S3A) prompted further investigation into the mechanism of CENP-A assembly at the S6 stage. S6 spermatocytes have a large nucleus (>20 μm in diameter) with three chromatin territories: the X-Y chromosomes pair together with the fourth chromosome pair to form one territory, while the second and third chromosome pairs form an additional two territories (Cenci et al., 1994).

**Fig. 3.**
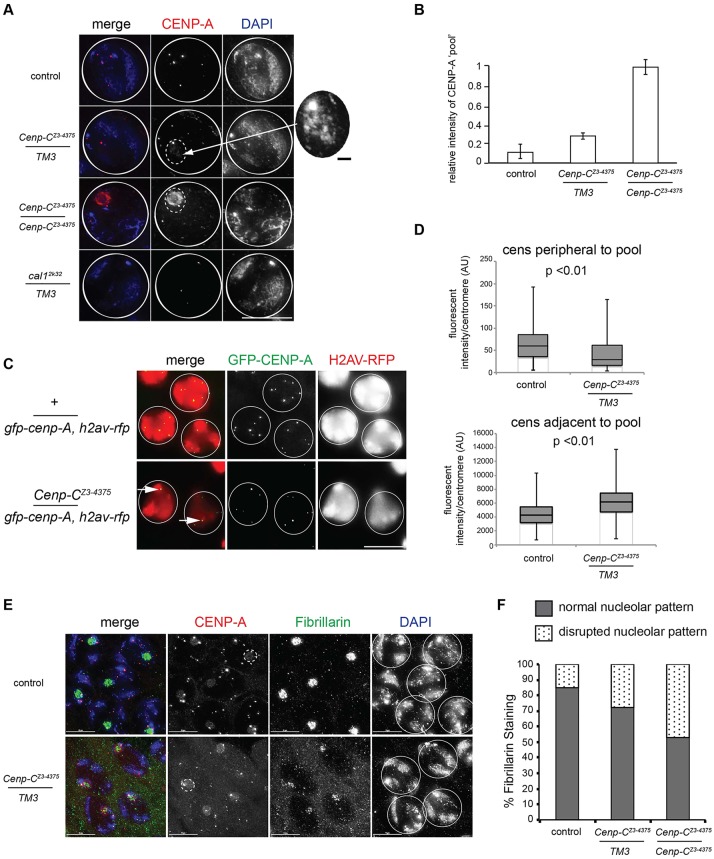
***Cenp-C^Z3-4375^* spermatocytes accumulate CENP-A in nucleoli.** (A) Control, *Cenp-C^Z3-4375^* and *cal1^2k32^* S6 spermatocytes fixed and stained with antibodies against CENP-A (red). DNA is stained with DAPI (blue). Solid white circle outlines S6 nuclei; dashed circle outlines the CENP-A pool; inset shows the CENP-A pool in the *Cenp-C^Z3-4375^* heterozygote with increased contrast. (B) Quantitation of the CENP-A pool (fluorescence intensity/nucleus; *n*=100) in S6 spermatocytes in control and *Cenp-C^Z3-4375^* testes. Error bars indicate s.e.m. (C) Live imaging of S5/6 *+/gfp-cid, h2av-rfp* and *Cenp-C^Z3-4375^/gfp-cid, h2av-rfp* spermatocytes. GFP-CENP-A is in green and H2AV-RFP in red. Arrows mark centromeres with high GFP-CENP-A intensity in *Cenp-C^Z3-4375^* spermatocytes. (D) Quantitation of CENP-A signal (fluorescence intensity/centromere) peripheral (*n*=300) or adjacent (*n*=140) to the CENP-A pool in control and *Cenp-C^Z3-4375^/TM3* prophase I spermatocytes (*t*-test). (E) Control and *Cenp-C^Z3-4375^/TM3* S6 spermatocytes fixed and immunostained for CENP-A (red) or Fibrillarin (green) and stained for DNA. Solid white circle outlines S6 nuclei; dashed circle outlines CENP-A pool. (F) Quantitation of normal versus disrupted Fibrillarin staining in control and *Cenp-C^Z3-4375^* S6 spermatocytes (*n*=70). Scale bars: 15 µm in A,C,E; 1 µm in inset in A.

Upon closer examination of *Cenp-C^Z3-4375^* S6 spermatocytes, although CENP-A intensity at most centromeres was reduced, we observed one or two bright CENP-A foci associated with a subnuclear ‘pool’ of CENP-A (Fig. 3A). Quantification revealed that a low-intensity CENP-A pool is detectable in most (Fig. 3B, see error bars) wild-type S6 spermatocytes, and that this is enhanced 2.5-fold in *Cenp-C^Z3-4375^* heterozygotes, with a further 3-fold increase in intensity in *Cenp-C^Z3-4375^* homozygotes (Fig. 3B). Live imaging of *Cenp-C^Z3-^*^4375^ S5/6 spermatocytes expressing GFP-CENP-A, and H2AV-RFP to mark chromatin, confirmed that most CENP-A foci were reduced in intensity and that one or two CENP-A foci remained visible (*n*=20, 70% cells; Fig. 3C). However, we could not detect a GFP-CENP-A pool by live imaging of *Cenp-C^Z3-4375^* spermatocytes, possibly as it is very dynamic.

Next, we quantified CENP-A intensity at centromeres either adjacent or peripheral to the CENP-A pool (Fig. 3D). This revealed that in *Cenp-C^Z3-4375^* mutants CENP-A foci adjacent to the pool had higher intensities while CENP-A foci peripheral to the pool had lower intensities compared with wild type (*P*<0.01, *t*-test). To exclude the possibility that the measured increase in CENP-A intensity was due to enhanced centromere clustering, we confirmed that there was no significant difference in the number of detectable CENP-A foci in *Cenp-C^Z3-4375^* S6 nuclei compared with controls (Fig. S3B). Rather, we observed a significant drop in the number of centromeres associated with the pool in *Cenp-C^Z3-4375^* mutants (Fig. S3C), suggesting defective centromere clustering/tethering at nucleoli, as reported in females (Unhavaithaya and Orr-Weaver, 2013).

We next aimed to locate the subnuclear CENP-A pool. Immunostaining of S6 spermatocytes for the nucleolar marker Fibrillarin confirmed that CENP-A localises to nucleoli in control and *Cenp-C^Z3-4375^* spermatocytes (Fig. 3E). We noted that Fibrillarin staining was disrupted in ∼30% of *Cenp-C^Z3-4375^* heterozygous spermatocytes, and that this was further disrupted in *Cenp-C^Z3-4375^* homozygotes (Fig. 3E,F, Fig. S3D), hinting that nucleolar integrity is disrupted when CENP-C function is compromised (see below). We conclude that *Cenp-C^Z3-4375^* spermatocytes that accumulate a pool of CENP-A in nucleoli are defective in CENP-A assembly at some centromeres in prophase I.

### X and Y centromeres at nucleoli assemble high levels of CENP-A in *Cenp-C* mutants

**Fig. 4.**
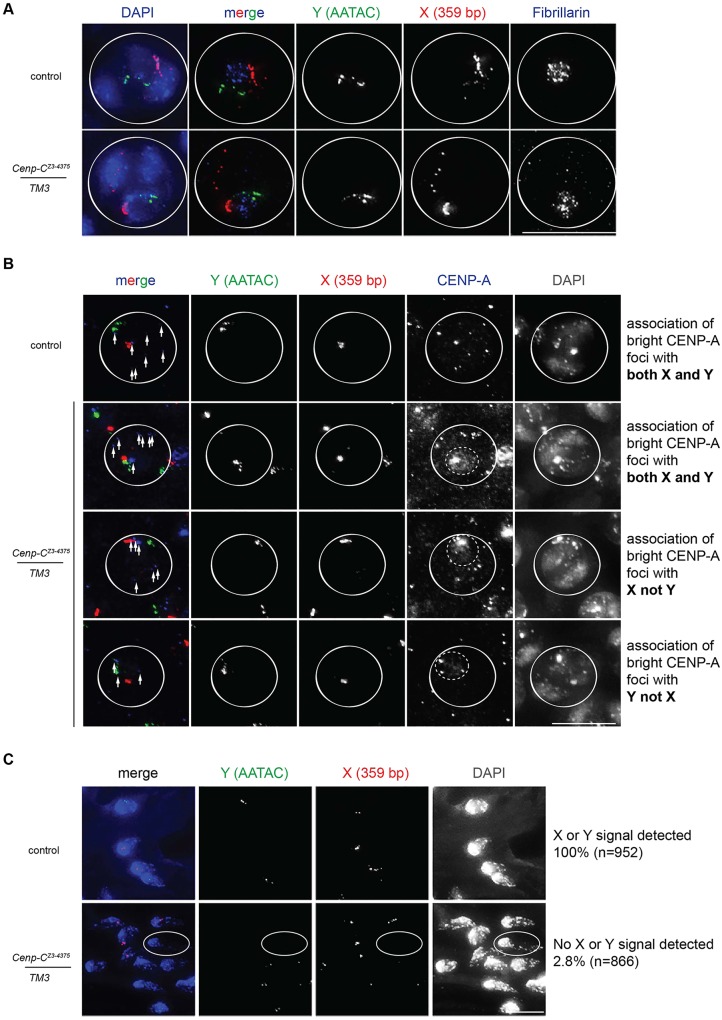
**X and Y centromeres at nucleoli assemble CENP-A in *Cenp-C^Z3-4375^* mutants.** (A) FISH for sequences specific for Y (AATAC, green) and X (359 bp, red) chromosomes, co-stained with antibodies against Fibrillarin (blue), performed on control and *Cenp-C^Z3-4375^/TM3* S5/6 spermatocytes. White circle outlines S5/S6 nuclei. (B) FISH for Y (green) and X (red) chromosomes, co-stained with antibodies against CENP-A (blue) and DNA is stained with DAPI (grey), performed on control and *Cenp-C^Z3-4375^/TM3* S5 spermatocytes. Solid white circle outlines S5 nuclei; dashed circle marks CENP-A pool; arrows mark detectable centromeres. (C) FISH for Y (green) and X (red) chromosomes performed on control and *Cenp-C^Z3-4375^/TM3* T5+ spermatids. DNA is stained with DAPI (blue). Spermatid lacking X and Y signal is circled. Percentage refers to pooled data from two experiments. Scale bars: 20 µm in A; 15 µm in B; 10 µm in C.

We next investigated which centromeres/chromosomes show enhanced CENP-A assembly in *Cenp-C^Z3-4375^* spermatocytes at prophase I. Given that rDNA loci are located on X and Y chromosomes (McKee et al., 2012), we hypothesized that X and Y centromeres associate with nucleoli and might aberrantly assemble CENP-A. Using fluorescent *in situ* hybridisation (FISH) probes specific for repeat sequences on the X (359 bp satellite) or Y (AATAC satellite) chromosomes (Tsai et al., 2011), and co-immunostaining for Fibrillarin, we confirmed that X and Y chromosomes associate with nucleoli in wild-type control and *Cenp-C^Z3-4375^/TM3* spermatocytes (Fig. 4A).

We next performed FISH with X and Y probes combined with anti-CENP-A immunostaining (Fig. 4B). In control spermatocytes, we observed four major staining patterns: brightest CENP-A foci associated with X and Y probes (57.3%, shown); brightest CENP-A foci associated with X but not Y probes (25.2%); brightest CENP-A foci associated with Y but not X probes (14.7%); or all CENP-A foci of equal intensity (2.6%) (Table 2). In *Cenp-C^Z3-4375^/TM3* spermatocytes, we also observed the brightest CENP-A foci associated with both X and Y probes, but at a lower frequency than wild type (36.1%), whereas the frequency of spermatocytes with brightest CENP-A foci associated with only X (30.6%) or only Y (31.1%) probes increased (Fig. 4B, Table 2). We conclude that in *Cenp-C^Z3-4375^* spermatocytes, X and Y centromeres adjacent to nucleoli frequently assemble aberrantly high levels of CENP-A.

**Table 2.**
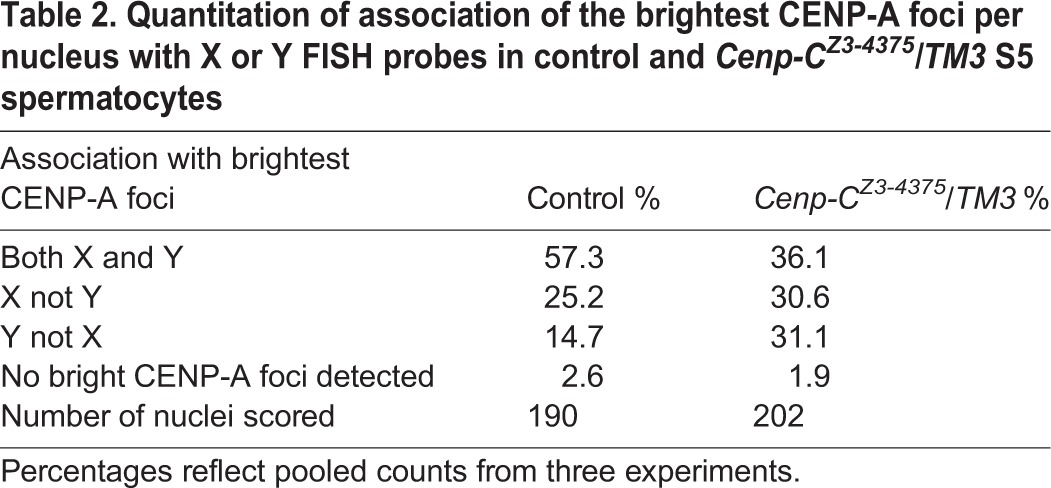
**Quantitation of association of the brightest CENP-A foci per nucleus with X or Y FISH probes in control and *Cenp-C^Z3-4375^/TM3* S5 spermatocytes**

We examined whether an enhanced CENP-A level on the X and Y centromeres might impact sex chromosome segregation in meiosis I. However, using FISH for X or Y probes combined with tubulin staining of meiotic spindles and DAPI staining of DNA, we found that the X and Y chromosomes segregate as expected in meiosis I in *Cenp-C^Z3-4375^* spermatocytes (not shown). Instead, analysis of T5+ spermatids revealed that 2.8% (*n*=866) of *Cenp-C^Z3-4375^/TM3* spermatids were negative for X or Y signals (Fig. 4C), a phenomenon never observed in wild-type spermatids and which might reflect sex chromosome loss, fragmentation or abnormal chromatin condensation in *Cenp-C^Z3-4375^* mutants (see Fig. 1E).

### Nucleolar integrity is disrupted in *Cenp-C* mutant spermatocytes at prophase I

**Fig. 5.**
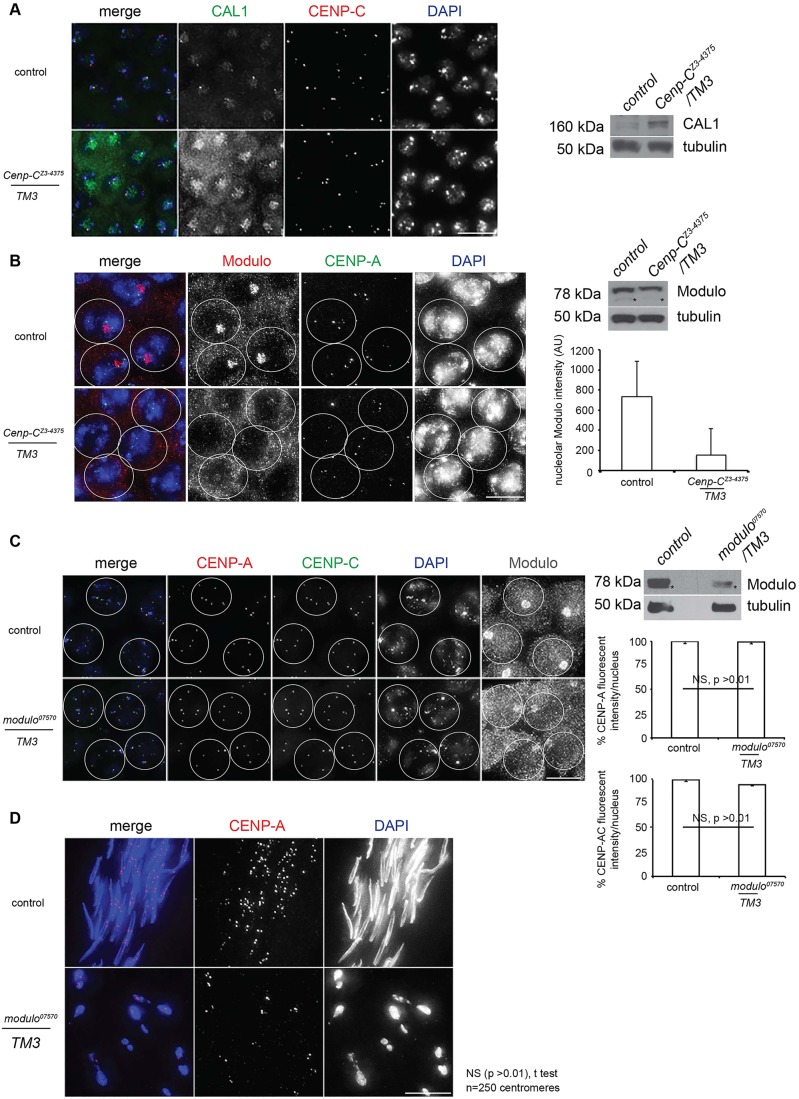
**Nucleolar CAL1 is elevated in *Cenp-C^Z3-4375^* spermatocytes, whereas Modulo is reduced.** (A) (Left) Control and *Cenp-C^Z3-4375^/TM3* prophase I spermatocytes (S3/4) fixed and stained with antibodies against CAL1 (green) and CENP-C (red). DNA is stained with DAPI (blue). (Right) Western blot of total extracts from control and *Cenp-C^Z3-4375^/TM3* larval testes probed with anti-CAL1 antibody and anti-tubulin antibody as loading control. (B) (Left) Control and *Cenp-C^Z3-4375^/TM3* S6 spermatocytes fixed and stained with antibodies against Modulo (red), CENP-A (green) or for DNA. White circle outlines S6 nuclei. (Right, top) Western blot of total extracts from control and *Cenp-C^Z3-4375^/TM3* larval testes probed with anti-Modulo antibody and anti-tubulin antibody as loading control. Asterisk marks a cross-reacting band. (Right, bottom) Quantitation of Modulo intensity (nucleolar fluorescence intensity/nucleus) in control and *Cenp-C^Z3-4375^/TM3* S6 spermatocytes (*n*=25)*.* Error bars indicate s.d. (C) (Left) Control and *modulo^07570^/TM3* S6 spermatocytes fixed and stained with antibodies against CENP-A (red), CENP-C (green), Modulo (grey) and for DNA (blue). White circle outlines S6 nuclei. (Right, top) Western analysis of Modulo expression in control and *modulo^07570^/TM3* testes, with anti-tubulin loading control. (Right, bottom) Quantitation of CENP-A or CENP-C (fluorescence intensity/nucleus; *n*=30) in control and *modulo^07570^/TM3* S6 spermatocytes. Error bars indicate s.e.m. NS, not significant (*t*-test). (D) Differentiating spermatids in control and *modulo^07570^/TM3* testes fixed and immunostained for CENP-A (red) and stained for DNA (blue). Scale bars: 10 µm in A,D; 15 µm in B,C.

We next wanted to gain further insight into the accumulation of meiotic CENP-A in nucleoli. As CAL1/HJURP localises to centromeres and nucleoli (Dunleavy et al., 2009; Erhardt et al., 2008; Foltz et al., 2009), we examined CAL1 localisation in *Cenp-C^Z3-4375^* testes (Fig. 5A). We focused on spermatocytes in early prophase I (S2/3), as centromeric CAL1 levels are reduced by prophase S6 (Dunleavy et al., 2012; Raychaudhuri et al., 2012). CAL1 immunostaining revealed that both nucleolar and centromeric CAL1 levels were increased in *Cenp-C^Z3-4375^* heterozygotes at prophase S2/3 (Fig. 5A). Western blotting of total (Fig. 5A) or fractionated (Fig. S3E) extracts from *Cenp-C^Z3-4375^* larval testes confirmed this increase, mostly in nucleoplasmic CAL1.

Given that Fibrillarin staining was perturbed in *Cenp-C^Z3-4375^* spermatocytes (Fig. 3E,F), we investigated further links between nucleolar integrity and CENP-A assembly in meiosis. To confirm nucleolar disruption, we stained *Cenp-C^Z3-4375^* heterozygous testes for a second nucleolar marker, Modulo (fly nucleolin), which functions in rDNA transcription and rRNA maturation (Ginisty et al., 1999). Immunostaining *Cenp-C^Z3-4375^/TM3* testes for Modulo revealed reduced staining compared with the control (Fig. 5B, Fig. S3F). Western analysis of total extracts from larval testes confirmed reduced Modulo protein in the *Cenp-C^Z3-4375^* mutant (by 76%; *P*<0.05, *t*-test), as did quantitation of nucleolar Modulo signal intensity (Fig. 5B).

As Modulo forms a complex with CAL1 and was previously implicated in CENP-A assembly in cultured *Drosophila* S2 cells (Chen et al., 2012), we next examined whether the observed defects in meiotic CENP-A assembly were due to reduced Modulo. We stained testes from *modulo^07570^/TM3* males for CENP-A, CENP-C and Modulo (Fig. 5C). However, in *modulo^07570^/TM3* S6 spermatocytes depleted for Modulo (depletion confirmed by immunoblotting and immunostaining with anti-Modulo), CENP-A and CENP-C levels were unaffected (Fig. 5C), indicating that prophase I centromere assembly is not perturbed. DAPI staining of *modulo^07570^/TM3* adult testes showed that spermatid differentiation was highly perturbed, consistent with a semi-sterile phenotype (Castrillon et al., 1993). However, despite gross morphological defects, CENP-A foci were still visible on fragmented spermatids at levels comparable to wild type (Fig. 5D).

We conclude that in *Cenp-C^Z3-4375^* testes, in addition to CENP-A, CAL1 accumulates in nucleoli. Nucleolar dense fibrillar (Fibrillarin) and granular (Modulo) components are disrupted/reduced in *Cenp-C* mutants; however, Modulo does not appear to be crucial for meiotic CENP-A assembly.

### RNA polymerase I transcription is required for CENP-A assembly in meiotic prophase I

We next tested if the transcriptional activity of nucleoli is disrupted in *Cenp-C^Z3-4375^* and *cal1^2k32^* mutants, and if transcription might impact CENP-A assembly. We first assayed the global transcription rate in testes by incorporation of the uridine analogue ethynl uridine (EU), a biosynthetic RNA label (Fig. S4A). In control spermatocytes, a bright EU signal enriched at rDNA in nucleoli was observed. Surprisingly, we observed reduced EU incorporation into *Cenp-C^Z3-4375^* and *cal1^2k32^* spermatocytes, indicating reduced transcription. Furthermore, introduction of YFP-tagged CENP-C in *Cenp-C^Z3-4375^* mutants led to a significant increase in EU incorporation (Fig. S4B).

**Fig. 6.**
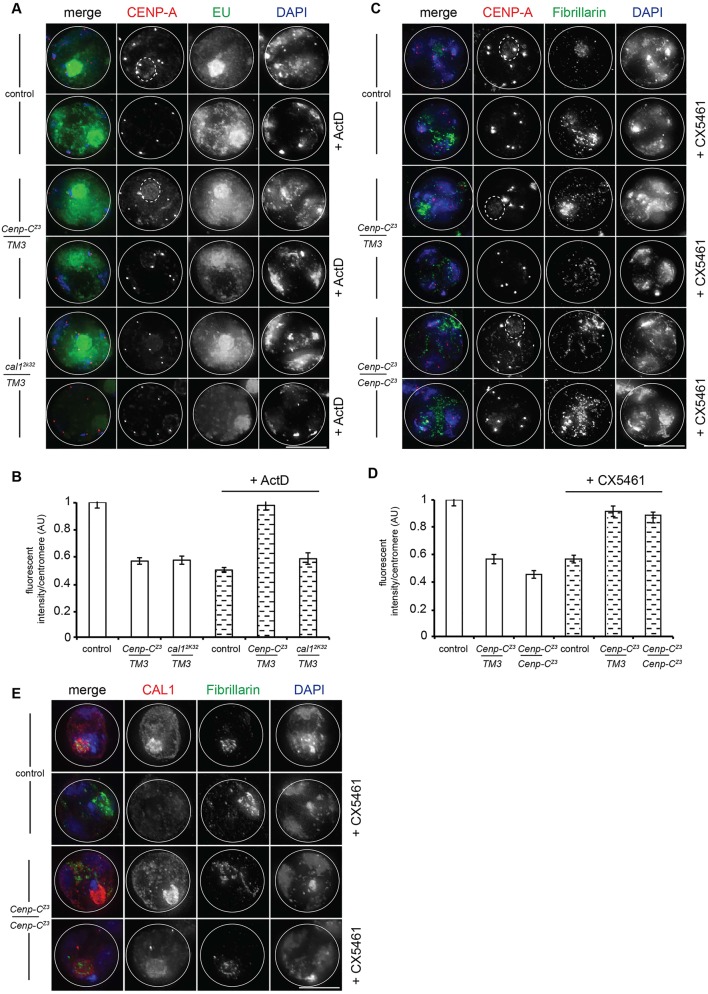
**Transcriptional activity is required for meiotic CENP-A assembly.** (A) Control, *Cenp-C^Z3-4375^/TM3* and *cal1^2k32^/TM3* S6 spermatocytes treated or untreated with actinomycin D (ActD), followed by pulse labelling with EU (green) and staining with antibodies against CENP-A (red). DNA is stained with DAPI (blue). (B) Quantitation of CENP-A (fluorescence intensity/‘peripheral’ centromere; *n*=300) in control, *Cenp-C^Z3-4375^/TM3* and *cal1^2k32^/TM3* S6 spermatocytes treated or untreated with ActD. Error bars indicate s.e.m. (C) Control, *Cenp-C^Z3-4375^/TM3* and *Cenp-C^Z3-4375^*/*Cenp-C^Z3-4375^* S6 spermatocytes treated or untreated with CX5461, stained with antibodies against CENP-A (red) and Fibrillarin (green). DNA is blue. (D) Quantitation of CENP-A (fluorescence intensity/‘peripheral’ centromere; *n*=100) in control, *Cenp-C^Z3-4375^/TM3* and *Cenp-C^Z3-4375^*^/*Z3-4375*^ S6 spermatocytes untreated or treated with CX5461. Error bars indicate s.e.m. (E) Control and*Cenp-C^Z3-4375^* homozygous S6 spermatocytes treated or untreated with CX5461 stained with antibodies against CAL1 (red) and Fibrillarin (green). DNA is blue. Scale bars: 10 µm.

To show that transcription is dependent on RNA polymerase (Pol) I and/or II, we treated larval testes with actinomycin D (ActD) at a concentration expected to inhibit both polymerases, followed by pulse labelling with EU (Fig. 6A). In spermatocytes treated with ActD, EU staining was consistently reduced, dispersed throughout the nucleus and no longer enriched in nucleoli, all indicators that the drug treatment was partially effective (Jao and Salic, 2008). To monitor effects of nucleolar transcription on centromere assembly, we also stained ActD-treated spermatocytes for CENP-A (Fig. 6A). Quantitation of control spermatocytes revealed a significant reduction (*P*<0.001, *t*-test) in CENP-A intensity at ‘peripheral’ centromeres, i.e. those located in chromosomal territories away from nucleoli upon treatment with ActD (Fig. 6B), suggesting that active transcription is normally required for CENP-A assembly. Additionally, we observed that ActD treatment of control spermatocytes perturbed centromere tethering to nucleoli (Fig. S4C). Strikingly, *Cenp-C^Z3-4375^/TM3* spermatocytes treated with ActD showed a significant increase (*P*<0.001, *t*-test) in CENP-A intensity at ‘peripheral’ centromeres (Fig. 6B) that was comparable to the untreated level, indicating rescue of CENP-A assembly. We did not observe such an increase in CENP-A intensity in *cal1^2k32^/TM3* spermatocytes (Fig. 6B), presumably as there is insufficient functional CAL1/CENP-C.

Next we confirmed that the observed rescue in CENP-A assembly in *Cenp-C^Z3-4375^* spermatocytes was specifically due to Pol I inhibition, using the SL1-specific inhibitor CX5461 (Drygin et al., 2011). Similar to ActD treatment, *Cenp-C^Z3-4375^* heterozygous and homozygous spermatocytes treated with CX5461 showed a significant recovery in CENP-A assembly (*P*<0.001, *t*-test), whereas control spermatocytes had reduced CENP-A intensity at centromeres (Fig. 6C,D), indicating that Pol I inhibition in *Cenp-C^Z3-4375^* spermatocytes rescues CENP-A assembly at ‘peripheral’ centromeres. Centromere tethering at nucleoli was again perturbed in control spermatocytes upon treatment with CX5461 (Fig. S4C), suggesting a specific requirement for Pol I transcription in centromere tethering at nucleoli. Quantitation of the CENP-A pool after treatment with CX5461 revealed a drop in intensity in control and *Cenp-C^Z3-4375^* mutants (Fig. S4D), and we confirmed that the drug was effective using Fibrillarin staining to quantify nucleolar disruption after treatment (Fig. S4E). Finally, CAL1 immunostaining revealed that control and *Cenp-C^Z3-4375^* homozygous S6 spermatocytes treated with CX5641 had reduced nucleolar CAL1 (Fig. 6E), suggesting that the observed rescue in *Cenp-C^Z3-4375^* was due to the release of CAL1 from nucleoli.

## DISCUSSION

Here we have analysed the effects of mutations in the key centromere assembly factors, *Cenp-C* and *cal1*, on *Drosophila* male meiosis. We show that a C-terminal CAL1 truncation (*cal1^2k32^*) and a C-terminal CENP-C missense mutation (*Cenp-C^Z3-4375^*) have dominant-negative effects on centromere function as well as fertility. Meiotic defects are consistent with errors reported after RNAi depletion of CENP-C and CAL1 in testes (Dunleavy et al., 2012; Raychaudhuri et al., 2012), as well as chromosome non-disjunction tests in *cal1^2k32^* females (Unhavaithaya and Orr-Weaver, 2013). However, our results suggest that CAL1 and CENP-C are particularly important for chromosome segregation in male meiosis, as phenotypes reported for *cal1^2k32^* and *Cenp-C^Z3-4375^* heterozygous females were comparably mild (Unhavaithaya and Orr-Weaver, 2013). Despite this, fertility in *cal1* and *Cenp-C* mutant females is even further reduced than in males, perhaps as far fewer gametes (oocytes) are normally produced in females. Also different from results in females were our findings that meiotic centromere (CENP-A) assembly/maintenance is perturbed in *cal1^2k32^* and *Cenp-C^Z3-4375^* males, pointing to potentially different mechanisms or dependencies between the sexes. CENP-A levels were lowest in *cal1^2k32^* testes, indicating that CAL1 is particularly important for meiotic CENP-A assembly and maintenance, whereas viable *Cenp-C^Z3-4375^* homozygotes, expressing mutant CENP-C at a low level, were competent for the first (albeit aberrant), but not second, phase of CENP-A assembly. Additionally, sufficient functional CENP-C is required for meiotic CENP-A maintenance. Roles for both CAL1 and CENP-C in CENP-A maintenance on mature sperm are particularly surprising given that neither is detectable at centromeres at this stage (Dunleavy et al., 2012), suggesting CAL1/CENP-C retention at low levels. Also surprising were findings that compromised CENP-C function impacts chromosome condensation and MEI-S332 localisation at prometaphase I, suggesting novel meiotic roles for CENP-C distinct from its role in kinetochore-microtubule attachment (Przewloka et al., 2011; Tanaka et al., 2009).

Our studies also reveal novel roles for centromere proteins in nucleolar integrity in meiotic prophase I. We find that localisation of the key nucleolar markers Fibrillarin and Modulo is disrupted in *Cenp-C^Z3-4375^* spermatocytes. Modulo was previously implicated in mitotic centromere assembly (Chen et al., 2012), yet it does not appear to play a major role in meiotic CENP-A assembly. We also discovered a nucleolar pool of CENP-A that is present at a low level in wild-type prophase I spermatocytes and enhanced 10-fold in *Cenp-C^Z3-4375^* homozygotes. Detection of the CENP-A pool in wild-type cells was variable, suggesting that the accumulation is transient, unstable or limited to a discrete S6 substage. CAL1, which is normally present in nucleoli in meiosis (Dunleavy et al., 2012; Raychaudhuri et al., 2012), also accumulates at high levels in nucleoli of *Cenp-C^Z3-4375^* spermatocytes. We presume that this increase is due to a compromised interaction between the C-terminal domain of CENP-C and CAL1 (Schittenhelm et al., 2010) and, as a result, CENP-A and CAL1 are ‘trapped’ in nucleoli. Intriguingly, upon RNAi depletion of CAL1 or CENP-A in testes, CENP-C accumulates in nucleoli of S6 spermatocytes (Dunleavy et al., 2012), suggesting that CENP-C might transiently associate with nucleoli to release CENP-A/CAL1. Furthermore, although rescue experiments in *Cenp-C^Z3-4375^* and *cal1^2k32^* mutants restored CENP-A levels at centromeres, we unexpectedly observed a high-intensity CENP-A pool (Fig. S2B). This suggests that as the CENP-A assembly pathway is partially restored in mutants, nucleolar CENP-A also accumulates. We also show that transcription is required for meiotic CENP-A assembly. Specifically, Pol I inhibition is sufficient to trigger CENP-A assembly in *Cenp-C^Z3-4375^* mutants, presumably as the accumulated nucleolar CAL1–CENP-A pool is released. Taken together, we propose that active Pol I transcription and CENP-C normally regulate the release of CAL1–CENP-A from nucleoli and that sequestering CAL1 to nucleoli limits CENP-A assembly until the correct time in the cell cycle. Another intriguing finding is that despite the disrupted nucleoli in *Cenp-C* mutants, CENP-A forms a pool that appears to have a distinct structure. One possibility is that CENP-A is housed in a nucleolar substructure not marked by Fibrillarin or Modulo. Finally, in addition to previous findings that centromere function is required for centromere tethering at nucleoli in females (Unhavaithaya and Orr-Weaver, 2013), we now show that centromere tethering to nucleoli in males is dependent on active transcription.

Also striking are findings in *Cenp-C^Z3-4375^* spermatocytes that centromeres located close to the nucleolar CENP-A pool assemble a high level of CENP-A, whereas centromeres away from nucleoli fail to assemble CENP-A. These results allow us to speculate that meiotic centromere assembly might occur via two separable pathways: (1) CAL1 delivers CENP-A to centromeres (conventional); and (2) centromeres assemble CENP-A if located adjacent to CAL1 and CENP-A in nucleoli (non-conventional). The non-conventional pathway might exist to ensure CENP-A assembly at some centromeres, e.g. sex chromosomes adjacent to nucleoli. Indeed, we show that X and Y centromeres usually have more CENP-A than other centromeres, although our results differ from a previous report of a 2-fold higher CENP-A level only on the Y centromere (Raychaudhuri et al., 2012). However, our use of FISH probes to identify X and Y chromosomes, combined with antibody staining of endogenous CENP-A, could explain discrepancies between the two studies. It is also possible that the high CENP-A observed on sex chromosomes is sometimes due to their close association with the small fourth chromosome (Tsai et al., 2011), which is also located proximal to nucleoli. Interestingly, we find that in *Cenp-C* mutants the highly elevated CENP-A at X or Y centromeres is not retained past prophase I, i.e. we did not observe abnormally bright CENP-A foci at M4/M5, M10/M11, T1 or T5 stages and CENP-A levels drop at all centromeres as meiosis progresses. This suggests that in *Cenp-C* mutants, although assembled in prophase, CENP-A is not stably incorporated.

This study is the first demonstration of specific requirements for centromere proteins in male fertility in *Drosophila*. The link between mutations in centromere proteins such as CENP-C, HJURP and CENP-A and fertility in humans seems probable, but has not yet been explored. This is also the first demonstration of the role of nucleolar CAL1. Whether nucleolar HJURP functions similarly in meiosis or mitosis in humans is not known, but given that mitotic CENP-A complexes contain nucleolar proteins, this might be the case. Intriguingly, pre-nucleosomal CENP-A is detected in nucleoli in human cells (Jansen et al., 2007). Indeed, consistent with a model proposed for mitotic centromere assembly (Chen et al., 2012), CENP-A assembly might be coupled to nucleolar disassembly at the end of meiotic prophase.

## MATERIALS AND METHODS

### *Drosophila* stocks

Flies were grown at 20°C on standard medium. *Cenp-C^Z3-4375^* and *cal1^2k32^* fly lines were gifts from T. Orr-Weaver (Unhavaithaya and Orr-Weaver, 2013). The fly line expressing GFP-CENP-A was provided by J. Lipsick (Wen et al., 2008), GFP-CENP-A, H2AV-RFP by S. Heidmann (Schuh et al., 2007), GFP-CAL1 and YFP-CENP-C by C. Lehner (Schittenhelm et al., 2010). The *modulo^07570^* line (#11795) was purchased from the Bloomington Stock Center. *y^+^ ry^+^* flies were used as wild type. For fertility tests, 2- to 3-day-old virgin males and females were crossed at 20°C and all progeny were counted upon eclosion.

### Cell biology

Testes were squashed, frozen in liquid nitrogen and fixed using methanol/acetone (Cenci et al., 1994) or 4% paraformaldehyde (PFA) for 10 min. Primary antibodies diluted in PBS containing 0.4% Triton X-100 and 1% BSA were incubated overnight at 4°C. Secondary antibody (Alexa Fluor conjugates, Molecular Probes) incubations were for 1 h at room temperature. For FISH, testes were fixed in PFA, frozen and passed through a cold ethanol series; probes based on Tsai et al. (2011) were directly labelled with Alexa Fluor conjugates (Eurofins; AATAC-488 for Y, 359 bp-546 for X) and 10 ng was used overnight at 20°C.

Testes were incubated with 300 nM CX5461 (Millipore) or 2 µg/ml actinomycin D (Sigma) for 30 min, followed by EU labelling (1 mM EdU, 30 min). For total protein extracts, 50 larval testes were lysed in 300 mM NaCl. Chromatin and nuclear soluble protein extracts were isolated from 100 larval testes by hypotonic extraction of nuclei, followed by lysis in 300 mM NaCl and centrifugation at 4000 ***g***. Live imaging of larval testes was performed as previously described (Dunleavy et al., 2012).

### Antibodies

For immunostaining, the following antibodies were used: rabbit anti-CENP-A (CID) antibody (Active Motif 39719; 1:500), guinea pig anti-CENP-C (Erhardt et al., 2008; 1:500), mouse anti-tubulin (Sigma DM1A; 1:100), chicken anti-Modulo (Mikhaylova et al., 2006; 1:200), mouse anti-Fibrillarin (Abcam ab4566; 1:500), guinea pig anti-MEI-S332 (Tang et al., 1998; 1:500) and rabbit anti-CAL1 (Bade et al., 2014; 1:100).

Western blotting employed the following antibodies: chicken anti-Modulo (Mikhaylova et al., 2006; 1:5000), rabbit anti-CENP-A (CID) (Lake Placid 39713; 1:1000), rabbit anti-CENP-C (Heeger et al., 2005; 1:8000), rabbit anti-CAL1 (Erhardt et al., 2008; 1:2000), mouse anti-Fibrillarin (Abcam ab4566; 1:1000), mouse anti-α-tubulin (Sigma B-5-1-2; 1:10,000), mouse anti-actin (Sigma AC-15; 1:5000) and rabbit anti-histone H3 (Millipore 17-10254; 1:50,000).

### Image acquisition, processing and quantification

Images were acquired using a DeltaVision Elite microscope system (Applied Precision). 20-30 *z*-sections at 0.2 µm were taken for each image at a constant exposure time. Raw images were deconvolved and projected using SoftWorx (Applied Precision) and uniformly scaled in Photoshop (Adobe). Quantification of fluorescence intensity at centromeres was performed using ImageJ software (NIH) on TIFF images after maximal intensity projections using a defined region of interest (ROI) with background correction.

### Statistics

*P*-values were calculated by two-tailed *t*-test, as stated with sample size (*n*) for each experiment/condition in the main text or figure legends.
